# Earthquake breakdown energy scaling despite constant fracture energy

**DOI:** 10.1038/s41467-022-28647-4

**Published:** 2022-02-22

**Authors:** Chun-Yu Ke, Gregory C. McLaskey, David S. Kammer

**Affiliations:** 1 School of Civil and Environmental Engineering, Cornell University, Ithaca, NY USA; 2 Institute for Building Materials, ETH Zürich, Zürich Switzerland

**Keywords:** Natural hazards, Seismology, Tectonics

## Abstract

In the quest to determine fault weakening processes that govern earthquake mechanics, it is common to infer the earthquake breakdown energy from seismological measurements. Breakdown energy is observed to scale with slip, which is often attributed to enhanced fault weakening with continued slip or at high slip rates, possibly caused by flash heating and thermal pressurization. However, seismologically inferred breakdown energy varies by more than six orders of magnitude and is frequently found to be negative-valued. This casts doubts about the common interpretation that breakdown energy is a proxy for the fracture energy, a material property which must be positive-valued and is generally observed to be relatively scale independent. Here, we present a dynamic model that demonstrates that breakdown energy scaling can occur despite constant fracture energy and does not require thermal pressurization or other enhanced weakening. Instead, earthquake breakdown energy scaling occurs simply due to scale-invariant stress drop overshoot, which may be affected more directly by the overall rupture mode – crack-like or pulse-like – rather than from a specific slip-weakening relationship.

## Abstract (web summary)

Earthquake breakdown energy is commonly interpreted as a proxy for fracture energy but is observed to scale with magnitude. Here the authors show that a scale-independent stress overshoot, as seen in the 3D dynamic earthquake rupture simulations, leads to comparable scaling despite constant fault fracture energy.

## Introduction

In an earthquake, strain energy stored elastically in the Earth’s crust is quickly transformed into radiated seismic waves, new fractures and wear products, and heat. This process is controlled, to a large extent, by the fault constitutive law, which describes how a fault’s strength evolves with slip or slip rate and how much energy is dissipated or available for continued rupture. Thus, the fault constitutive law and associated fault properties are key to better understand and possibly predict earthquake mechanics. However, these properties are difficult to measure in the Earth. A number of studies tried to constrain fault constitutive behavior using seismological observations of earthquakes, and, in particular, the way that earthquake parameters scale from small to large^1–4^. One significant seismological observation, known as the breakdown energy, is thought to be related to the slip weakening process, and is often assumed to be a proxy for fracture energy. However, the breakdown energy is frequently observed to be negative-valued^1,4,5^ and, if positive-valued, to scale with slip. Hence, larger earthquakes with more total slip appear to dissipate more energy (per unit rupture area) than small earthquakes.

If breakdown energy was equivalent to fracture energy, as often assumed (e.g., ref. ^6–8^), it is expected to be a material or interfacial property that is positive-valued and, for most engineering materials, observed to be only mildly dependent on the rupture propagation^8^. Hence, it is not expected to scale over many orders of magnitude as seismologically observed. Various theories have been developed over the years to explain the discrepancy of breakdown energy scaling. For instance, it was suggested that frictional weakening distance could increase with increasing earthquake size^1^. Other studies suggested additional mechanisms that activate as the earthquake rupture grows larger and slip distances increase such as thermal pressurization (e.g., ref. ^3,4,9,10^) or off-fault energy sinks (e.g., ref. ^11–14^). High-velocity friction experiments that show continued weakening with cumulative slip were also offered as support. However, the fracture energy measured from those experiments appears to plateau at around 2 m of slip^7^ while seismologically estimated breakdown energy of natural earthquakes continues beyond this limit and scales across all sizes of earthquakes^1^. Furthermore, none of these theories and observations provide an explanation for negative-valued breakdown energy. Hence, a fully consistent theory explaining all observations of seismologically inferred breakdown energy remains missing.

A running earthquake arrests either because the rupture front enters a region of the fault that is stronger or has a more stable rheology compared to the nucleation region, i.e., a barrier^9,10,15,16^, or the front enters a region with low initial stress and hence subcritical driving force^17^. While the first scenario implies a larger fault fracture energy for a larger earthquake rupture, the second scenario does not.

Here, we will show that the observed breakdown energy scaling does not require any complex or scale dependent fault constitutive law. We will demonstrate a simple but plausible scenario where constant fault friction but non-uniform initial stress results in the same scaling of breakdown energy. The non-uniform initial stress is not required to produce scaling of seismologically estimated breakdown energy^1^ ${G}^{\prime}$; a strong barrier model^18,19^ that results in stress overshoot (described below) would produce the same result. However, the non-uniform initial stress enables our models to have constant fracture energy over the entire domain. We solve the three-dimensional model with fully dynamic numerical simulations that employ linear slip-weakening friction. From the models, we determine the breakdown energy and other seismic source parameters. The result is unambiguous: breakdown energy does not correspond to the locally imposed constant fracture energy; it results from a scale-invariant stress overshoot. Stress overshoot occurs in crack-like ruptures when a fast-propagating rupture front arrests but parts of the fault continue to slip^19,20^. The implications of overshoot on breakdown energy were discussed in Abercrombie and Rice^1^ and dismissed for crack-like ruptures by Viesca and Garagash^4^. However, we argue that overshoot plays a central role in breakdown energy scaling and that our model results are consistent with current estimates of apparent stress and stress drop for real earthquakes.

## Results

### Numerical model

**Fig. 1:**
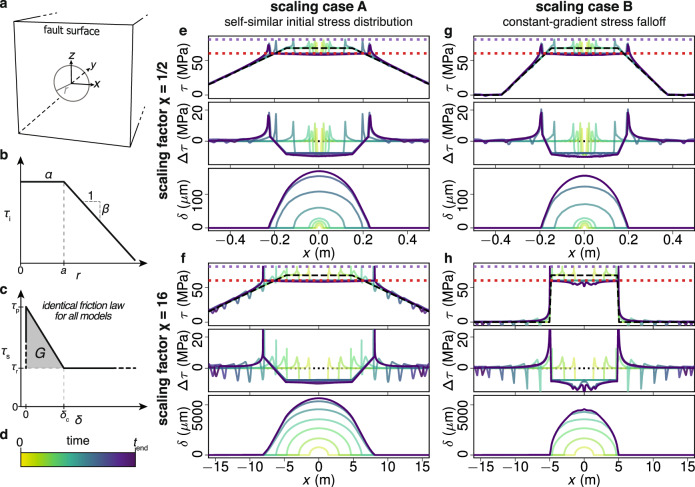
Nucleation and arrest of simulated earthquake at fault with non-uniform initial stress. **a** Schematics of the numerical model, where the fault surface is defined on the *xz* plane (*y* = 0) and embedded in a homogeneous elastic full space. **b** Parametric initial stress distribution where *α* is the initial stress amplitude at the plateau with radius *a* and *β* is the gradient of initial stress decrease outside the plateau. **c** The slip-weakening friction law where peak strength *τ*_p_, residual strength *τ*_r_, and critical slip distance *δ*_c_ are identical for all models. Fracture energy *G* is the shaded area. **d** The color map for curves in (**e**–**h**) that indicates the simulation time, where *t*_end_ is time of arrest. **e**–**h** Snapshots of stress *τ*, stress drop Δ*τ* and slip *δ* at *y* = *z* = 0 with *χ* = 2^−1^ and *χ* = 2^4^ in scaling case A and B, respectively. Purple and red dotted lines indicate *τ*_p_ and *τ*_r_ from the friction law (see c), respectively.

We consider a two-dimensional planar fault (*y* = 0) embedded in a three-dimensional isotropic elastic medium with shear modulus *μ* = 12 GPa and shear wave speed *c*_s_ = 2126 m/s (Fig. 1a). The fault is governed by a linear slip-weakening friction law (Fig. 1c) with peak strength *τ*_p_ = 80 MPa, residual strength *τ*_r_ = 60MPa and critical slip distance *δ*_c_ = 10^−5^ m. Hence, the fault is characterized by realistic stress levels for natural faults, and a well-defined and constant fracture energy *G* = 100 Jm^−2^, which is larger than estimates on smooth, precut faults^21^, but smaller than intact rocks^22^. The initial stress has uniform amplitude *α* = 67.5 MPa within a circular region centered at the origin. Earthquake ruptures are nucleated at the origin by a slowly increasing area of reduced fault strength (see Supplementary Note S1). While the nucleation process is artificial, it is sufficiently small and slow to not affect the unstable propagation of the earthquake that occurs spontaneously. After nucleation, the earthquake rupture velocity quickly accelerates to the Rayleigh wave speed where it remains constant^23,24^. The earthquake is nearly axisymmetric but grows slightly faster in the direction of the applied shear load (i.e., *x*–direction).

We explore two different scenarios for earthquake scaling by imposing an initial stress distribution that depends on scaling factor *χ*. Both scenarios reproduce the scaling of breakdown energy despite constant fracture energy, and produce identical initial rupture growth within a circular region of radius *a* = 0.3*χ* m, but they differ in the way they arrest, and this provides insight into the role that arrest plays in calculated source parameters. In scaling case A, initial stress decreases outside the circular region (Fig. 1b) at spatial rate *β* = 75/*χ* MPa/m (cf. Fig. 1e and Fig. 1f and note scaling of *x*–axis). In scaling case B, we impose a constant stress falloff *β* = 300 MPa/m (cf. Fig. 1g and Fig. 1h). The *χ* = 2^−2^ model is identical in both scaling cases. We highlight that the resolution of the numerical models, the nucleation procedure, and fault friction properties, including fracture energy *G* (Fig. 1c), are kept constant across all scales.

### Scaling of earthquake source properties

**Fig. 2:**
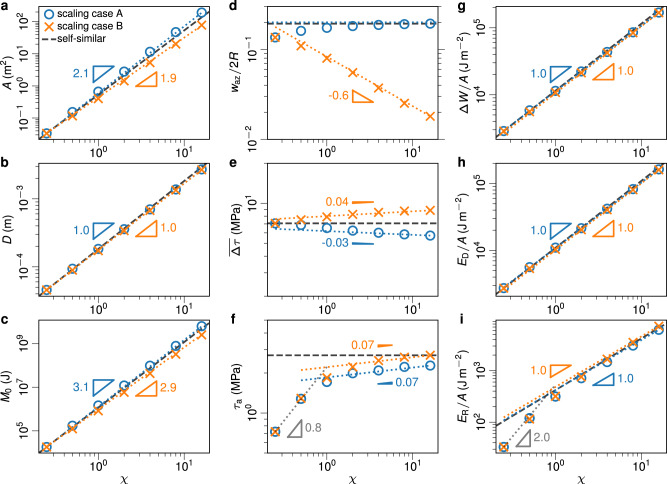
Scaling of earthquake source parameters in both scaling cases. Triangles and the annotated numbers indicate the power of trend lines, i.e., *p* in *V* ∝ *χ*^*p*^ for different parameters *V*. Black dash lines indicate ideal self-similar scaling relations. Scaling is shown for: **a** Earthquake rupture area *A.* **b** Average slip *D.* **c** Seismic moment *M*_0_. **d** Earthquake arrest zone width *w*_az_ scaled by rupture diameter 2*R.* **e** Averaged stress drop $\overline{{{\Delta }}\tau }$. **f** Apparent stress *τ*_a_. **g** Averaged total released strain energy Δ*W*/*A.* **h** Averaged dissipated energy *E*_D_/*A*. **i** Averaged radiated energy *E*_R_/*A*.

The scaling of calculated earthquake source properties is shown in Fig. 2, and definitions for these parameters in the context of our models are described below. Earthquake rupture area *A* is the area of the slipped fault patch, i.e., *A* = ∫_Σ_d*S* for Σ = {*x*, *z* ∈ *U*∣*δ*(*x*, *z*) > 0} and *U* is the entire simulation fault surface domain with *y* = 0. The spatially averaged slip distance *D* is the final slip *δ*_f_ averaged over the rupture area, i.e., *D* = ∫_Σ_*δ*_f_(*x*, *z*)d*S*/*A* with *δ*_f_(*x*, *z*) = *δ*(*x*, *z*, *t* = *t*_end_) (Fig. 1e–h). Moment release rate $\dot{M}(t)=\mu {\int}_{U}\dot{\delta }(x,z,t){{{{{{{\rm{d}}}}}}}}S$, where $\dot{\delta }={{{{{{{\rm{d}}}}}}}}\delta /{{{{{{{\rm{d}}}}}}}}t$ is the on-fault slip rate. The seismic moment is hence given by ${M}_{0}=\int\nolimits_{0}^{{t}_{{{{{{{{\rm{end}}}}}}}}}}\dot{M}(t){{{{{{{\rm{d}}}}}}}}t$. Alternatively, the seismic moment could be computed as *M*_0_ = *μAD*, which yields equivalent results.

The averaged stress drop $\overline{{{\Delta }}\tau }$ is one of the most important inferred earthquake source properties. A common seismological approach to determine $\overline{{{\Delta }}\tau }$ is to assume a uniform stress drop^25^ and estimate it from seismic source parameters using $\overline{{{\Delta }}\tau }=7{M}_{0}/16{R}^{3}$, where $R=\sqrt{A/\pi }$. Despite the simplification of assumed uniform stress drop, which is clearly not satisfied in our model (see Δ*τ* in Fig. 1e–h), the above formulation provides an accurate estimation of $\overline{{{\Delta }}\tau }$, and more sophisticated approaches^26,27^ do not lead to significantly different results (see Supplementary Note S2).

We observe that $\overline{{{\Delta }}\tau }$ is nearly scale-invariant in our model (Fig. 2e), where scaling case A and B bound closely a scale-invariant behavior, consistent with seismologically inferred measurements^28–31^. With the self-similar initial stress distribution of scaling case A, the arrest zone width^17^ *w*_az_ = *R* − *a* is a nearly constant percentage of the average rupture radius *R* (Fig. 2d). For scaling case A, initial stress decreases more gradually (smaller *β*) for larger earthquakes than for smaller ones, and *A* scales with *χ*^2.1^ rather than the expected *χ*^2.0^ because large earthquakes rupture proportionally further into unfavorably stressed regions. For this reason *M*_0_ scales with *χ*^3.1^ instead of *χ*^3.0^. However, the average slip *D* scales as *χ*^1.0^, therefore stress drop ($\sim\! D/\sqrt{A}$) decreases slightly (Fig. 2e). In scaling case B, *w*_az_/2*R* is smaller for larger earthquakes (Fig. 2d) and *A* scales as *χ*^1.9^ (rather than *χ*^2.0^) because larger and larger ruptures meet relatively steeper and steeper stress gradients and therefore rupture proportionally shorter and shorter distances into unfavorable stress. Similarly, *M*_0_ scales with *χ*^2.9^ and stress drop increases slightly with increasing earthquake size.

We also analyze the energy budget for our model earthquakes. Using the energy-considered averaged initial stress ${\overline{\tau }}_{{{{{{{{\rm{i}}}}}}}}}$ and final stress $\overline{\tau}_{{\rm{f}}}$^26,27^, the averaged total released strain energy is computed ${{\Delta }}W/A=\frac{1}{2}\left({\overline{\tau }}_{{{{{{{{\rm{i}}}}}}}}}+{\overline{\tau }}_{{{{{{{{\rm{f}}}}}}}}}\right)D$, which is a simplified formulation of the method proposed by Kostrov^32^ (see Fig. 2g and Methods). We also compute the dissipated energy *E*_D_ = *E*_H_ + *E*_G_, which combines heat *E*_H_ and total breakdown energy *E*_G_, by ${E}_{{{{{{{{\rm{D}}}}}}}}}=\int\nolimits_{0}^{{t}_{{{{{{{{\rm{end}}}}}}}}}}{\int}_{U}\tau (x,z,t)\dot{\delta }(x,z,t){{{{{{{\rm{d}}}}}}}}S{{{{{{{\rm{d}}}}}}}}t$ (Fig. 2h). Finally, we estimate the radiated energy *E*_R_ (see Methods and Supplementary Note S3), and we observe *E*_R_/*A* ∝ *D*^1^, consistent with a nearly constant apparent stress *τ*_a_ = *μE*_R_/*M*_0_ ≈ 2MPa (Fig. 2f, i; ref. ^5,28,33,34^).

From our scaling analysis, we conclude that our model reproduces the scaling behavior of all important seismic source properties. The expected scaling properties for self-similar rupture are either reproduced by the models or are bounded by scaling cases A and B (Fig. 2a–d). We specifically note that *A* ∝ *D*^2^, *M*_0_ ∝ *D*^3^, and $\overline{{{\Delta }}\tau }\propto {D}^{0}$, consistent with standard earthquake scaling^35^.

### Scaling of breakdown energy

**Fig. 3:**
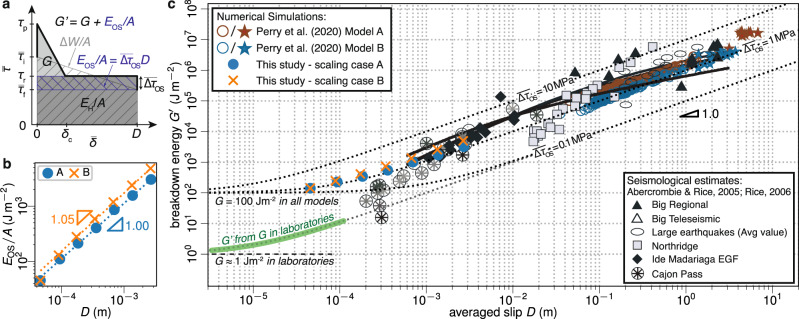
Scaling relations of seismologically estimated breakdown energy $${G}^{\prime}$$. **a** Schematics of energy partition on the averaged stress versus averaged slip ($$\overline{\tau }-$$$$\overline{\delta }$$) space, where the dark blue hashed area indicates the overestimation of fracture energy *G* by $${G}^{\prime}$$ due to stress overshoot $${\overline{{{\Delta }}\tau }}_{{{{{{{{\rm{OS}}}}}}}}}$$, i.e., final stress $${\overline{\tau }}_{{{{{{{{\rm{f}}}}}}}}}$$ being lower than residual stress *τ*_r_. **b** The averaged overshoot energy *E*_OS_/*A* (Eq. (3)) is equivalent to $${G}^{\prime}-G$$, which scales linearly with averaged slip *D*. **c** Comparison of $${G}^{\prime}$$ from our models to data by ref. ^1,3,9^. Three black dotted curves indicate $${G}^{\prime}$$ computed by Eq. (2) with *G* = 100 Jm^−2^. The gray dotted curve diverged from the $${\overline{{{\Delta }}\tau }}_{{{{{{{{\rm{OS}}}}}}}}}=0.1\,{{{{{{{\rm{MPa}}}}}}}}$$ curve at the lower end of *D* highlights the $${G}^{\prime}$$ expected in laboratories^21,56^ and demonstrates that *G* is the lower limit of $${G}^{\prime}$$ for $${\overline{{{\Delta }}\tau }}_{{{{{{{{\rm{OS}}}}}}}}}\ge 0$$.

The dissipated energy *E*_D_ is composed of the total breakdown energy *E*_G_ and heat *E*_H_ (Fig. 3a). The averaged breakdown energy $\overline{G}\approx {E}_{{{{{{{{\rm{G}}}}}}}}}/A$ is often interpreted as a proxy for the fracture energy *G*, which controls the dynamics of the fault rupture^23^, and hence is of great importance for seismology. Abercrombie and Rice^1^ proposed a seismological approach to estimate $\overline{G}$ by

1 $${G}^{\prime}=\frac{D}{2}\left(\overline{{{\Delta }}\tau }-\frac{2\mu {E}_{{{{{{{{\rm{R}}}}}}}}}}{{M}_{0}}\right)\,,$$

which has been widely used (e.g., ref. ^5,36–39^). We compute ${G}^{\prime}$ for our simulations following Eq. (1) and observe that our scaling cases A and B follow ${G}^{\prime}\propto {D}^{0.8}$ and ${G}^{\prime}\propto {D}^{1.0}$, respectively (Fig. 3c). Abercrombie and Rice^1^ found ${G}^{\prime}\propto {D}^{1.28}$. Their exponent of 1.28 is greater than 1 as a result of magnitude-dependent *τ*_a_ and $\overline{{{\Delta }}\tau }$, which they argued was significant, but has not been supported by more recent studies^5,28,33,34^. While the scaling in our models is not an exact match, which could be attributed to additional aforementioned dissipative mechanisms, we argue that it is within the uncertainty of the observational data (Fig. S11), and emphasize that ${G}^{\prime}$ scales orders of magnitude in our simulations even though the actual fault property of fracture energy *G* is kept constant (Fig. 3).

Using energy calculations proposed by previous studies^26,27^, we rewrite ${G}^{\prime}$ as

2 $${G}^{\prime}=G+{\overline{{{\Delta }}\tau }}_{{{{{{{{\rm{OS}}}}}}}}}D\,,$$

where ${\overline{{{\Delta }}\tau }}_{{{{{{{{\rm{OS}}}}}}}}}=\left({\tau }_{{{{{{{{\rm{r}}}}}}}}}-{\overline{\tau }}_{{{{{{{{\rm{f}}}}}}}}}\right)$ is the energy-considered averaged stress overshoot (see Fig. 3a and Methods). Hence, the breakdown energy consists of the sum of the fracture energy and the overshoot energy, which we define as

3 $${E}_{{{{{{{{\rm{OS}}}}}}}}}/A\equiv {\overline{{{\Delta }}\tau }}_{{{{{{{{\rm{OS}}}}}}}}}D\,.$$

In our simulations, the averaged stress overshoot ${\overline{{{\Delta }}\tau }}_{{{{{{{{\rm{OS}}}}}}}}}=1-2\,{{{{{{{\rm{MPa}}}}}}}}$ is positive and nearly scale-invariant (see Fig. S6 and Fig. S9). ${G}^{\prime}$ of large earthquakes with small *G* and scale invariant overshoot is dominated by the overshoot, hence: ${G}^{\prime}\propto {D}^{1}$ (Eq. (2)). In Fig. 3c, we show the results of Eq. (2) for different levels of overshoot (${\overline{{{\Delta }}\tau }}_{{{{{{{{\rm{OS}}}}}}}}}$) compared to seismological estimates of ${G}^{\prime}$ for earthquakes of various sizes, and find ${\overline{{{\Delta }}\tau }}_{{{{{{{{\rm{OS}}}}}}}}}\approx 1\,{{{{{{{\rm{MPa}}}}}}}}$. Further, *G* serves as a lower bound of ${G}^{\prime}$, as shown by the dotted curves at lower *D* in Fig. 3c. This implies the fault fracture energy is bounded by *G* ≤ 100 Jm^−2^. Finally, we note that a model with a strong barrier^18,19^ rather than an initial stress taper would present a similar scaling of ${G}^{\prime}$ due to a scale-invariant stress overshoot. Such a condition is most similar to scaling case B, where arrest occurs very abruptly, and hence it provides an upper bound for stress overshoot, which is ${\overline{{{\Delta }}\tau }}_{{{{{{{{\rm{OS}}}}}}}}}=0.2\overline{{{\Delta }}\tau }$ (see Fig. S9).

Abercrombie and Rice^1^ considered whether ${G}^{\prime}$ scaling could be dominated by overshoot, as we suggest, but ultimately dismissed it because their observed ${\tau }_{{{{{{{{\rm{a}}}}}}}}}/\overline{{{\Delta }}\tau }=0.1$ would then imply an overshoot of $0.4\overline{{{\Delta }}\tau }$. Such a large overshoot would exceed the $0.15\overline{{{\Delta }}\tau }$ to $0.2\overline{{{\Delta }}\tau }$ of the Madariaga^19^ model, which was considered an upper bound, due to its abrupt arrest. However, ${\tau }_{{{{{{{{\rm{a}}}}}}}}}/\overline{{{\Delta }}\tau }=0.1$ is somewhat lower than found elsewhere^40^ (${\tau }_{{{{{{{{\rm{a}}}}}}}}}/\overline{{{\Delta }}\tau }=0.33$). Our models produce ${\tau }_{{{{{{{{\rm{a}}}}}}}}}/\overline{{{\Delta }}\tau }=0.3-0.4$, which is within the range of seismic estimates, and our observed overshoot ($0.1\overline{{{\Delta }}\tau }$ to $0.2\overline{{{\Delta }}\tau }$) is consistent with both the Madariaga^19^ upper bound and the energy balance relation (ref. ^20^ Eq. 1c) used by Abercrombie and Rice^1^.

If our interpretation is correct, and *G* is indeed small (*G* ≤ 100 Jm^−2^), how can this be reconciled with estimates of *G* = 10^6^ Jm^−2^ derived from finite-fault kinematic inversions (e.g., ref. ^2^), or with laboratory data^13^ that indicates 1 m weakening distances? As for the kinematic inversions, we find that the minimum resolvable characteristic weakening length ${\hat{\delta }}_{{{{{{{{\rm{c}}}}}}}}}$ could be limited by bandwidth^41^. For example, assuming a typical 0.5 s smoothing operator (e.g., ref. ^2^), minimum resolvable *δ*_c_ is of order 500 mm. Assuming *τ*_p_ − *τ*_r_ = 10 MPa, this places minimum resolvable *G* = 2.5 MJ/m^2^. (See Supplementary Note S4, Fig. S4.) Considering the laboratory results, most of the observed weakening occurs while fault slip accelerates^13^, and 1 m weakening distances resulted from sluggish slip acceleration (6.5 m/s^2^) compared with measurements^42^ from dynamic rupture fronts (>20,000 m/s^2^). Experiments^43^ that imposed more abrupt loading (30 m/s^2^) exhibited far smaller weakening distances (0.02 m). Thus, large inferred weakening distances and therefore large *G* may be an artifact of laboratory loading procedures that are slow compared to the fault acceleration imposed by a dynamic rupture front during an earthquake.

Finally, our interpretation helps reconcile observations of ${G}^{\prime}\approx 0$ and negative ${G}^{\prime}$, found for numerous earthquakes^1,4^, yet rarely discussed. Such cases would result from negligible overshoot or undershoot i.e., ${\overline{\tau }}_{{{{{{{{\rm{f}}}}}}}}} \, > \, {\tau }_{{{{{{{{\rm{r}}}}}}}}}$, a condition typically associated with pulse-like earthquake ruptures^44^, where the fault is elastically reloaded after a short period of slip. Indeed, we find that a simple model with scale-invariant random overshoot that ranges between −1 and 2 MPa and negligibly small *G* produces ${G}^{\prime}\propto {D}^{1.0}$, fits the Abercrombie and Rice^1^ data reasonably well (see Fig. S11 and dotted curves in Fig. 3c), and produces a catalog where one-third of all events exhibit ${G}^{\prime}\le 0$, similar to the large earthquakes studied by Viesca and Garagash^4^. All in all, our simulations and proposed interpretation of ${G}^{\prime}$ reconciles the co-existence of breakdown energy scaling and negative ${G}^{\prime}$.

### Rupture growth and arrest from source time functions

**Fig. 4:**
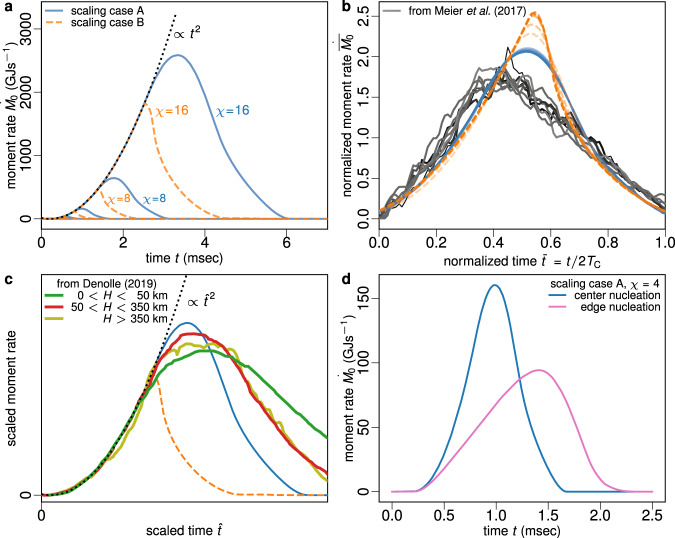
Source time function $\dot{M}(t)$ of numerical simulations and natural earthquakes. **a** All models follow an identical $\dot{M}(t)\propto {t}^{2}$ growth independent of scaling parameter *χ* until reaching unfavorable stress conditions. The black dotted curve is a quadratic fit for growth phase of moment rate history. **b** Normalized source time functions, where ${T}_{{{{{{{{\rm{C}}}}}}}}}=\int\nolimits_{0}^{\infty }t\dot{M}{{{{{{{\rm{d}}}}}}}}t\left/\int\nolimits_{0}^{\infty }\dot{M}{{{{{{{\rm{d}}}}}}}}t\right.$ and the area under $\dot{\overline{M}}(\overline{t})$ is 1. Gray curves are natural earthquakes with *M*_w_ ≥ 7 presented in ref. ^48^. **c** Scaled source time functions with matching growth phase. Green and red curves are natural earthquakes at various source depths *H* adapted from ref. ^47^. **d** Source time function of a rupture nucleated at the center of the stress plateau, and at the edge of the stress plateau.

We study the spontaneous growth and arrest of rupture in our dynamic model by comparing the earthquake source time function or moment rate function $\dot{M}(t)$ with natural observations. $\dot{M}(t)$ consists of three phases: (1) a self-similar growth phase (2) a divergence from self-similar growth near peak moment rate and (3) the post-peak decay. In the growth phase, we observe $\dot{M}(t)\propto {t}^{2}$, at all scales (see Fig. 4a), as expected for roughly circular earthquake ruptures propagating without bound^45,46^. Self-similar moment rate growth has also been observed for large earthquakes – in some cases $\dot{M}(t)\propto {t}^{2}$ (Fig. 4c; ref. ^47^) but in others $\dot{M}(t)\propto {t}^{1}$ (Fig. 4b; ref. ^48^). Near peak $\dot{M}(t)$, earthquake rupture begins to arrest and the boundaries to rupture growth are increasingly felt. Scaling case B produces earthquakes that arrest far too quickly compared to observations^47,48^. Scaling case A’s gradual arrest is a reasonable fit to observations, however, all of our models exhibit rapid post-peak decay of $\dot{M}(t)$ and negative skew, while natural earthquakes show a more gradual post-peak behavior and slightly positive skew^47,48^. This suggests that our simulated earthquakes do not arrest slowly enough or that slip ceases too quickly after the rupture initially begins to arrest.

To better understand the above discrepancies, we compared the previously described simulations, where nucleation occurs in the center of the circular region of favorable initial stress, to a case where the earthquake nucleates close to the edge of the favorably stressed region, i.e., (*x*, *y*, *z*) = (0.7*a*, 0, 0.7*a*), denoted “edge nucleation” (see Fig. S10). The latter case quickly reaches unfavorable initial stress on NE side and then ruptures unilaterally to the SW. The difference in the source time function is striking (Fig. 4d). The edge nucleation quickly diverges from the $\dot{M}(t)\propto {t}^{2}$ self-similar growth curve and instead $\dot{M}(t)$ grows nearly linearly. The growth phase is about 50 percent longer than in the symmetric case, though the decay is very similar.

While the edge nucleation model does not reconcile the abbreviated post-peak behavior (and actually makes the skewness worse), it offers a satisfying explanation for linear growth rate. The earliest part of the growth phase, when unbounded growth is expected, is difficult to resolve from kinematic models^48^. Proper resolution of early growth would require fault plane discretization size to depend on the distance from the hypocenter^49^. When normalized source time functions are plotted on a linear scale, as shown in Fig. 4b (or ref. ^48^ Fig. 2b–d), their form is dominated by the final increase in moment rate just before rupture arrest exceeds rupture growth. In this stage, rupture growth will, on average, be bounded on at least one side, and will produce the nearly linear increase in moment rate demonstrated by our edge nucleation model.

## Discussion

Previous work assumed that breakdown energy ${G}^{\prime}$ was a proxy for fracture energy *G*, and was therefore dominated by the way strength evolved with slip. Our modeling offers an alternative interpretation. It demonstrates that scaling of ${G}^{\prime}$ can arise naturally from the dynamics of rupture growth and arrest, and it can occur even with constant *G* and does not require a scale-dependent constitutive behavior such as enhanced weakening at large fault slip. As noted by Abercrombie and Rice^1^, ${G}^{\prime}$ depends strongly on overshoot and undershoot, which are affected by the rupture type (crack versus pulse) and the manner in which an earthquake arrests. Our observation of ${G}^{\prime}$ scaling is directly linked to the fact that our model exhibits scale-independent stress overshoot. This also suggests that negative ${G}^{\prime}$ is an indication of undershoot^44^, which is thought to occur for self-healing slip pulses, while the majority of earthquakes overshoot^20^, a property of crack-like ruptures. However, our models’ overshoot-induced scaling (${G}^{\prime}\propto {D}^{0.8}$ and ${G}^{\prime}\propto {D}^{1.0}$) is somewhat weaker than the ${G}^{\prime}\propto {D}^{1.28}$ observed by Abercrombie and Rice^1^. Our model produces a self-similar source time function and offers an explanation for the seismologically observed linear growth phase^48^, but decays too quickly compared to natural observations.

## Methods

### Parametrization of initial stress distribution

The on-fault initial stress distribution *τ*_i_(*x*, *y* = 0, *z*) is applied in the x-direction and is parameterized as

M1 $${\tau }_{{{{{{{{\rm{i}}}}}}}}}(r)=\alpha -\beta (r-a)H(r-a)\,,$$

where $r=\sqrt{{x}^{2}+{z}^{2}}$ is the distance to the origin, *H* is the Heaviside step function, *a* is the radius of the stress plateau with amplitude *α*, and *β* is the spatial-rate of stress decay outside the stress plateau (Fig. 1b).

### Numerical method

Numerical simulations are conducted with our implementation of the spectral boundary integral (SBI) method^50^ to solve for a fully dynamic interface debonding problem. The method is based on previous methodological studies^51–53^ and the implementation has been verified through comparison with SCEC-USGS dynamic rupture code verification examples^54^. The SBI method has inherent periodic boundary conditions at the boundaries of the simulated fault domain, *i.e*., (*x*, *y*, *z*) = (±*L*/2, 0, ± *L*/2) in domain {*x* ∈ [−*L*/2, *L*/2);*y* = 0; *z* ∈ [−*L*/2, *L*/2)}. We verified that the simulated domain is large enough that the rupture and the reflected waves do not affect the ruptured region within the simulation duration. Ruptures were nucleated by a seed crack at the center of the fault, in which the peak strength is manually decreased to *τ*_r_ and extended at 10% of the Rayleigh wave speed (see Supplementary Text S1).

### Seismic source parameters

*M*_0_ calculated through integration over $\dot{M}(t)=\mu {\int}_{U}\dot{\delta }(x,z,t){{{{{{{\rm{d}}}}}}}}S$ is theoretically identical to *M*_0_ = *μAD*. We have verified that their results are the same as the amplitude at the low-frequency plateau of the moment rate spectrum

M2 $${{\Omega }}(f)={{{{{{{\mathcal{F}}}}}}}}(\dot{M}(t))\,,$$

where ${{{{{{{\mathcal{F}}}}}}}}$ denotes Fourier transformation.

### Energy Calculations

The total strain energy released from an arrested earthquake rupture can be integrated solely on the ruptured domain Σ^32^:

M3 $${{\Delta }}W=\frac{1}{2}{\int}_{{{\Sigma }}}\left[{\tau }_{{{{{{{{\rm{i}}}}}}}}}(x,z)+{\tau }_{{{{{{{{\rm{f}}}}}}}}}(x,z)\right]{\delta }_{{{{{{{{\rm{f}}}}}}}}}(x,z){{{{{{{\rm{d}}}}}}}}S\,,$$

which can be rewritten into a simpler form with the energy-considered averaging method^26,27^:

M4 $${{\Delta }}W/A=\frac{1}{2}\left({\overline{\tau }}_{{{{{{{{\rm{i}}}}}}}}}^{{{{{{{{\rm{E}}}}}}}}}+{\overline{\tau }}_{{{{{{{{\rm{f}}}}}}}}}^{{{{{{{{\rm{E}}}}}}}}}\right)D\,,$$

where ${\overline{\tau }}^{{{{{{{{\rm{E}}}}}}}}}$ is the average stress weighted by the final slip

M5 $${\overline{\tau }}^{{{{{{{{\rm{E}}}}}}}}}(t)=\frac{{\int}_{{{\Sigma }}}\tau (x,z,t){\delta }_{{{{{{{{\rm{f}}}}}}}}}(x,z){{{{{{{\rm{d}}}}}}}}S}{{\int}_{{{\Sigma }}}{\delta }_{{{{{{{{\rm{f}}}}}}}}}(x,z){{{{{{{\rm{d}}}}}}}}S}\,.$$

Therefore, the energy-considered averaged stress drop is simply

M6 $${\overline{{{\Delta }}\tau }}^{{{{{{{{\rm{E}}}}}}}}}={\overline{\tau }}_{{{{{{{{\rm{i}}}}}}}}}^{{{{{{{{\rm{E}}}}}}}}}-{\overline{\tau }}_{{{{{{{{\rm{f}}}}}}}}}^{{{{{{{{\rm{E}}}}}}}}}\,,$$

where $${\overline{\tau }}_{{{{{{{{\rm{i}}}}}}}}}^{{{{{{{{\rm{E}}}}}}}}}={\overline{\tau }}^{{{{{{{{\rm{E}}}}}}}}}(t=0)$$ and $${\overline{\tau }}_{{{{{{{{\rm{f}}}}}}}}}^{{{{{{{{\rm{E}}}}}}}}}={\overline{\tau }}^{{{{{{{{\rm{E}}}}}}}}}(t={t}_{{{{{{{{\rm{end}}}}}}}}})$$.

We consider two approaches for radiated energy. First, we compute ${E}_{{{{{{{{\rm{R}}}}}}}}}^{{{{{{{{\rm{N}}}}}}}}}$ from integrated on-fault stress and slip evolution. As shown by ref. ^32^ and Appendix C in ref. ^55^, *E*_R_ can be conveniently evaluated numerically from the result of numerical simulation through

M7 $${E}_{{{{{{{{\rm{R}}}}}}}}}^{{{{{{{{\rm{N}}}}}}}}}=\frac{1}{2}{\int}_{U}\left[{\tau }_{{{{{{{{\rm{f}}}}}}}}}(x,z)-{\tau }_{{{{{{{{\rm{i}}}}}}}}}(x,z)\right]{\delta }_{{{{{{{{\rm{f}}}}}}}}}(x,z){{{{{{{\rm{d}}}}}}}}S-\!\!\int\nolimits_{0}^{{t}_{{{{{{{{\rm{end}}}}}}}}}}{\int}_{U}\left[\tau (x,z,t)-{\tau }_{{{{{{{{\rm{i}}}}}}}}}(x,z)\right]\dot{\delta }(x,z,t){{{{{{{\rm{d}}}}}}}}S{{{{{{{\rm{d}}}}}}}}t\,.$$

This approach is equivalent to an energy conservation equation (following ref. ^1^)

M8 $${E}_{{{{{{{{\rm{R}}}}}}}}}^{{{{{{{{\rm{C}}}}}}}}}={{\Delta }}W-{E}_{{{{{{{{\rm{D}}}}}}}}}\,,$$

if the *τ*_i_ term in the second integration of Eq. (M7) is reorganized into

M9 $$\int\nolimits_{0}^{{t}_{{{{{{{{\rm{end}}}}}}}}}}{\int}_{U}{\tau }_{{{{{{{{\rm{i}}}}}}}}}(x,z)\dot{\delta }(x,z,t){{{{{{{\rm{d}}}}}}}}S{{{{{{{\rm{d}}}}}}}}t={\int}_{U}{\tau }_{{{{{{{{\rm{i}}}}}}}}}(x,z){\delta }_{{{{{{{{\rm{f}}}}}}}}}(x,z){{{{{{{\rm{d}}}}}}}}S\,,$$

and moved into the first term. Both approaches yield similar results with some differences for small *χ*, which we associate to small ruptures arresting before reaching Rayleigh wave speed and the effects of rupture initiation process in our numerical simulation. We use ${E}_{{{{{{{{\rm{R}}}}}}}}}^{{{{{{{{\rm{N}}}}}}}}}$ to represent *E*_R_ due to its superior numerical stability (see Supplementary Text S3).

Here we start from the energy conservation equation divided by *A* on both sides,

M10 $${{\Delta }}W/A={\overline{G}}^{\prime}+{E}_{{{{{{{{\rm{H}}}}}}}}}/A+{E}_{{{{{{{{\rm{R}}}}}}}}}/A\,,$$

where Δ*W* is the total strain energy released, *A* is rupture area, ${\overline{G}}^{\prime}$ is the spatially averaged breakdown energy, *E*_H_ is heat, and *E*_R_ is radiated energy. Replacing ${{\Delta }}W/A=\frac{1}{2}\left({\overline{\tau }}_{{{{{{{{\rm{i}}}}}}}}}^{{{{{{{{\rm{E}}}}}}}}}+{\overline{\tau }}_{{{{{{{{\rm{f}}}}}}}}}^{{{{{{{{\rm{E}}}}}}}}}\right)D$ ^26,27^, ${E}_{{{{{{{{\rm{H}}}}}}}}}/A={\overline{\tau }}_{{{{{{{{\rm{f}}}}}}}}}^{{{{{{{{\rm{E}}}}}}}}}D$, and $A={M}_{0}/\left(\mu D\right)$, the equation becomes

M11 $$\frac{1}{2}\left({\overline{\tau }}_{{{{{{{{\rm{i}}}}}}}}}^{{{{{{{{\rm{E}}}}}}}}}+{\overline{\tau }}_{{{{{{{{\rm{f}}}}}}}}}^{{{{{{{{\rm{E}}}}}}}}}\right)D={\overline{G}}^{\prime}+{\overline{\tau }}_{{{{{{{{\rm{f}}}}}}}}}^{{{{{{{{\rm{E}}}}}}}}}D+{E}_{{{{{{{{\rm{R}}}}}}}}}\frac{\mu D}{{M}_{0}}\,.$$

With some rearrangements and replacing ${\overline{\tau }}_{{{{{{{{\rm{i}}}}}}}}}^{{{{{{{{\rm{E}}}}}}}}}-{\overline{\tau }}_{{{{{{{{\rm{f}}}}}}}}}^{{{{{{{{\rm{E}}}}}}}}}={\overline{{{\Delta }}\tau }}^{{{{{{{{\rm{E}}}}}}}}}$, ${\overline{G}}^{\prime}$ can be expressed as

M12 $${\overline{G}}^{\prime}=\frac{1}{2}\left({\overline{{{\Delta }}\tau }}^{{{{{{{{\rm{E}}}}}}}}}-\frac{2\mu {E}_{{{{{{{{\rm{R}}}}}}}}}}{{M}_{0}}\right)D\,,$$

which takes a very similar form as ${G}^{\prime}$ (Eq. (1)). The deduction above actually assumes that ${\overline{G}}^{\prime}$ is

M13 $${\overline{G}}^{\prime}={\int}_{U}\int\nolimits_{0}^{{\delta }_{{{{{{{{\rm{f}}}}}}}}}}\left(\tau -{\tau }_{{{{{{{{\rm{f}}}}}}}}}\right){{{{{{{\rm{d}}}}}}}}\delta {{{{{{{\rm{d}}}}}}}}S/A\,$$

by the definition of ${E}_{{{{{{{{\rm{H}}}}}}}}}/A={\overline{\tau }}_{{{{{{{{\rm{f}}}}}}}}}^{{{{{{{{\rm{E}}}}}}}}}D={E}_{{{{{{{{\rm{D}}}}}}}}}/A-{\overline{G}}^{\prime}$, where ${E}_{{{{{{{{\rm{D}}}}}}}}}={\int}_{U}\int\nolimits_{0}^{{\delta }_{{{{{{{{\rm{f}}}}}}}}}}\tau {{{{{{{\rm{d}}}}}}}}\delta {{{{{{{\rm{d}}}}}}}}S$ is the dissipated energy. Whereas the fracture energy should be the area above *τ*_r_,

M14 $$G={\int}_{U}\int\nolimits_{0}^{{\delta }_{{{{{{{{\rm{f}}}}}}}}}}\left(\tau -{\tau }_{{{{{{{{\rm{r}}}}}}}}}\right){{{{{{{\rm{d}}}}}}}}\delta {{{{{{{\rm{d}}}}}}}}S/A\,.$$

Similar to ref. ^1^, when assuming *G* and *τ*_r_ are constants, ${\overline{G}}^{\prime}$ can be expressed as

M15 $${\overline{G}}^{\prime}=G+\left({\tau }_{{{{{{{{\rm{r}}}}}}}}}-{\overline{\tau }}_{{{{{{{{\rm{f}}}}}}}}}^{{{{{{{{\rm{E}}}}}}}}}\right)D\,.$$

Note that the difference between ${\overline{G}}^{\prime}$ and *G* involves the final-slip-weighted-average final stress ${\overline{\tau }}_{{{{{{{{\rm{f}}}}}}}}}^{{{{{{{{\rm{E}}}}}}}}}$, *i.e*.,

M16 $${\overline{\tau }}_{{{{{{{{\rm{f}}}}}}}}}^{{{{{{{{\rm{E}}}}}}}}}=\frac{{\int}_{{{\Sigma }}}{\tau }_{{{{{{{{\rm{f}}}}}}}}}(x,z){\delta }_{{{{{{{{\rm{f}}}}}}}}}(x,z){{{{{{{\rm{d}}}}}}}}S}{{\int}_{{{\Sigma }}}{\delta }_{{{{{{{{\rm{f}}}}}}}}}(x,z){{{{{{{\rm{d}}}}}}}}S}\,,$$

and the stress overshoot $\left({\tau }_{{{{{{{{\rm{r}}}}}}}}}-{\tau }_{{{{{{{{\rm{f}}}}}}}}}(x,z)\right)$ in our models appear to be larger at locations with larger slip, as shown in Supplementary Fig. S7. This highlights the spatially averaged stress overshoot cannot be used when evaluating the accuracy of ${G}^{\prime}$, as the effect of stress overshoot is clearly amplified by its correlation with larger slip.

## Supplementary information

Supplementary Information

## Data Availability

The simulation data generated in this study have been deposited in the ETH Research Collection database under accession code ethz-b-000527677 [10.3929/ethz-b-000527677].

## Code availability

The code used in the current study is available on a public GitLab repository [https://gitlab.com/uguca/projects/breakdown_energy_scaling].
