# Supplementary material for: Earthquake breakdown energy scaling despite constant fracture energy
Source: Nat Commun. 2022 Feb 22;13:1005. doi: 10.1038/s41467-022-28647-4 (PMC8863786; doi:10.1038/s41467-022-28647-4)
Supplement: Supplementary file 1 — Supplementary Information [file 41467_2022_28647_MOESM1_ESM.pdf]

Supplementary Information for "Earthquake Breakdown Energy Scaling Despite Constant Fracture Energy"

Chun-Yu Ke[1], Gregory C. McLaskey[1], David S. Kammer[2]

[1] *School of Civil and Environmental Engineering, Cornell University, Ithaca, New York, USA*

[2] *Institute for Building Materials, ETH Zürich, Zürich, Switzerland*

Contents of this file

1. Supplementary Notes S1 to S4
2. Figures S1 to S11

Introduction

To investigate the scaling of seismologically estimated breakdown energy[1] G', we started from the length scale of laboratory earthquakes in our previous studies[2,3] and scaled the results with numerical models in two different scaling cases with identical interfacial properties. Here, we provide details of the numerical approaches (Supplementary Note S1 and Fig. S1), different methods of averaged stress drop calculation (Supplementary Note S2 and Fig. S2), different methods of radiated energy calculation (Supplementary Note S3 and Fig. S3), the relations between sampling frequency f_s and the minimum resolvable critical slip distance δ_c (Supplementary Note S4 and Fig. S4), scaling relations of seismologically estimated breakdown energy G' with different

approaches (Fig. S5), scaling relations of stress overshoot $(\tau_\mathrm{r} - \overline{\tau}_\mathrm{f}^\mathrm{E})$ (Fig. S6), an example of the spatial distribution of stress τ, stress change $\Delta\tau$, stress overshoot $(\tau_\mathrm{r} - \overline{\tau}_\mathrm{f}^\mathrm{E})$, and slip δ over time (Fig. S7-S8), stress overshoot normalized by static stress drop (Fig. S9), a comparison between simulations with center nucleation versus edge nucleation (Fig. S8), and an example of randomly generated pseudo-earthquakes (Fig. S11) overlayed on the observations of ref.[1].

Supplementary Note S1. Numerical simulations

Ruptures were nucleated by a seed crack at the center of the fault, in which the peak strength τ_p was manually decreased to the residual strength τ_r and extended at 10% of the Rayleigh wave speed. The effective fracture energy $G = \frac{1}{2}(\tau_\mathrm{p} - \tau_\mathrm{r})\delta_\mathrm{c}$ is 0 within the seed crack since $\tau_\mathrm{p} = \tau_\mathrm{r}$. The growth of the seed crack is stopped when its radius reaches a certain prescribed limit to avoid further affecting the fault behavior. This limit was chosen to be slightly larger than the critical nucleation length through linear elastic fracture mechanics theory[4]. All of the models in this study initiate identically from within the constant initial stress region ($r < a$), with identical critical nucleation length $L_c = \frac{16}{\pi}\frac{\mu(\lambda+\mu)}{(\lambda+2\mu)}\frac{G}{(\tau_\mathrm{i}-\tau_\mathrm{r})^2} \approx 0.072$ m.

The element size is identical for all the models in this study and was chosen through the trade-off between accuracy (smaller element size) and maximum achievable χ (larger element size). For a fixed domain $\{x \in [-L/2, L/2); y = 0; z \in [-L/2, L/2)\}$, we uniformly discretized the domain with element size $\Delta x = \Delta z = L/N_x = L/N_z = \sqrt{N}$, where $N = N_x \times N_z$ is the total number of elements and $L = 2\chi$ m. At $\chi = 2^{-2}$, we analyzed the convergence of all the parameters of interest, *i.e.*, A, D, M_0, $\overline{\Delta\tau}$, f_c, $w_\mathrm{az}/2R$, $\overline{\Delta\tau}$ (see Supplementary Note S2), $\Delta W/A$,

E_D/A, and E_R/A (see Supplementary Note S3), as shown in Fig. S1. With consideration of the range of χ, we finally selected element size of $1/128 = 0.0078\ \mathrm{m}$ in both x and z directions. Note that this convergence study was done with a set of models with initial stress τ_i, strengths τ_p and τ_r, and δ_c that were all 10x smaller than the values in the simulations shown in the manuscript (and closer to the laboratory values). However, the two sets of models did not required separate convergence studies since they have identical critical nucleation length $L_c =$ and cohesive zone width[5] $w_\mathrm{coh} = \frac{9\pi}{32E}\frac{G}{(\tau_\mathrm{i}-\tau_\mathrm{r})^2}$ with $G = \frac{1}{2}(\tau_\mathrm{p} - \tau_\mathrm{r})\delta_\mathrm{c}$. The two sets of models also exhibited nearly identical scaling relations.

Supplementary Note S2. Averaged stress drop

We define the spatial distribution of static stress drop $\Delta\tau(x,z)$ as

$$\Delta\tau(x,z) = \tau_\mathrm{i}(x,z) - \tau_\mathrm{f}(x,z)\ , \tag{S1}$$

where $\tau_\mathrm{i}(x,z) = \tau(x,z,t=0)$ is the initial stress distribution and $\tau_\mathrm{f}(x,z) = \tau(x,z,t=t_\mathrm{end})$ is the final stress distribution. By assuming the stress drop is uniform within the ruptured area[6], the seismologically estimated average stress drop $\overline{\Delta\tau}^\mathrm{S}$ can be calculated through seismic source parameters by

$$\overline{\Delta\tau}^\mathrm{S} = \frac{7}{16}\frac{M_0}{R^3}\ , \tag{S2}$$

where $R = \sqrt{A/\pi}$ is the source radius of a circular rupture with area A. $\overline{\Delta\tau}^\mathrm{S}$ has been widely-used as a first-order estimation of stress drop. However, the underlying uniform stress drop assumption is not always valid, especially for the models in scaling case A considered in this study. The spatial average stress drop over the whole ruptured area is the most straightforward averaging scheme and

can be expressed as

$$\overline{\Delta\tau}^{\mathrm{A}} = \frac{1}{A}\int_{\Sigma} \Delta\tau(x,z) dS \ . \tag{S3}$$

However, this quantity is highly affected by the heterogeneity of the rupture, *e.g.*, $\Delta\tau_{\mathrm{i}}(x,z)$ in our models, and has been shown mostly irrelevant to the fault constitutive law[7]. Fig. S1f shows that the estimation of $\overline{\Delta\tau}^{\mathrm{A}}$ is not converged for the selected element size. We believe it is mainly due to the final rupture size of the $\chi = 2^{-2}$ model is not large enough. Thus, the error should be quickly diminished for larger models. As shown in Fig. S2, all three different estimates of $\overline{\Delta\tau}$ are nearly scale-independent.

Supplementary Note S3. Radiated Energy

As shown by Kostrov[8] and Appendix C in ref.[9], the radiated energy can be computed through time history of shear stress and slip rate at the fault plane,

$$E_{\mathrm{R}}^{\mathrm{N}} = \frac{1}{2}\int_{U} \left[\tau_{\mathrm{f}}(x,z) - \tau_{\mathrm{i}}(x,z)\right] \delta_{\mathrm{f}}(x,z)\,\mathrm{d}S - \int_{0}^{t_{\mathrm{end}}}\int_{U} \left[\tau(x,z,t) - \tau_{\mathrm{i}}(x,z)\right] \dot{\delta}(x,z,t)\,\mathrm{d}S\,\mathrm{d}t \ . \tag{S4}$$

Considering $\int_0^{t_{\mathrm{end}}} \tau_{\mathrm{i}}(x,z)\dot{\delta}(x,z,t)\,\mathrm{d}t = \tau_{\mathrm{i}}(x,z)\delta_{\mathrm{f}}(x,z)$, the equation above can also be rearranged into

$$E_{\mathrm{R}}^{\mathrm{N}'} = \frac{1}{2}\int_{U} \left[\tau_{\mathrm{f}}(x,z) + \tau_{\mathrm{i}}(x,z)\right] \delta_{\mathrm{f}}(x,z)\,\mathrm{d}S - \int_{0}^{t_{\mathrm{end}}}\int_{U} \tau(x,z,t)\dot{\delta}(x,z,t)\,\mathrm{d}S\,\mathrm{d}t \ . \tag{S5}$$

Other than $E_{\mathrm{R}}^{\mathrm{N}}$ and $E_{\mathrm{R}}^{\mathrm{C}}$, we also considered a seismological approach for E_{R} estimation. Following Eqn. 16 in ref.[10], E_{R} can be estimated through seismic moment rate by

$$E_{\mathrm{R}}^{\mathrm{S}} = \frac{4\pi}{5\rho c_{\mathrm{s}}^{5}}\int_{0}^{\infty} f^2\Omega^2(f)\,\mathrm{d}f \ , \tag{S6}$$

where ρ is the density of the surrounding medium and c_s is the shear-wave speed.

Fig. S3 shows estimations of E_R computed through different methods. E_R^C and $E_\mathrm{R}^{\mathrm{N}'}$ yield very close estimations. However, they are actually less accurate than E_R^N according to the convergence study in Supplementary Note S1, as shown in Fig. S1i. All estimates scale similarly, with a slight deviation in E_R^N and E_R^S at lower χ, as shown in Fig. S3. We believe that this deviation was caused by the area of the seed crack described in Supplementary Note S1 is relatively large, and the rupture front has not fully accelerated to the Rayleigh wave speed before it reaches unfavorable stress conditions and starts to arrest. E_R^S is systematically higher than other estimates for $\chi > 1/2$ and is therefore not considered in further discussions.

Supplementary Note S4. Local-point approach of breakdown energy estimation

Theoretically, the breakdown energy at a specific point within the rupture area can be accurately estimated through

$$G(\tau(\delta)) = \int_0^{\delta_\mathrm{f}} (\tau(\delta) - \tau_\mathrm{min})\,\mathrm{d}\delta \ , \tag{S7}$$

if the transient responses in $\tau(t)$ and $\delta(t)$ are faithfully recorded or recovered through kinetic inversion approaches, and τ over/undershoots are corrected for. Averaging procedures[7] could be applied but require corrections for energy partition and remain imperfect[11] estimations for G. Even when the local-point approach is correctly applied, overestimation on G can still occur. Assuming the sampling rate of both $\delta(t)$ and $\tau(t)$ measurements is f_s, consider an extreme scenario that one sample was done right on the timing of τ reaching τ_p and the next sample was done when τ first

dropped to τ_r. The measured location slipped δ_c during the time difference between two samples $\Delta t = 1/f_\mathrm{s}$, *i.e.*, the averaged slip rate $\dot{\delta} = \delta_\mathrm{c} f_\mathrm{s}$. If the fault accelerated instantaneously and slipped at a constant rate $\dot{\delta}_\mathrm{max}$, then the minimum resolvable δ_c at a specific f_s and $\dot{\delta}_\mathrm{max}$ will be

$$\delta_\mathrm{c} = \dot{\delta}_\mathrm{max}/f_\mathrm{s} \ , \tag{S8}$$

as shown in Fig. S4b. To demonstrate how low sampling rate affects the estimation of G and δ_c, we down-sample $\delta(t)$ and $\tau(t)$ from our simulations and show that the area associated with the fracture energy increases (Fig. S4a). This concept is also discussed in ref.[12].

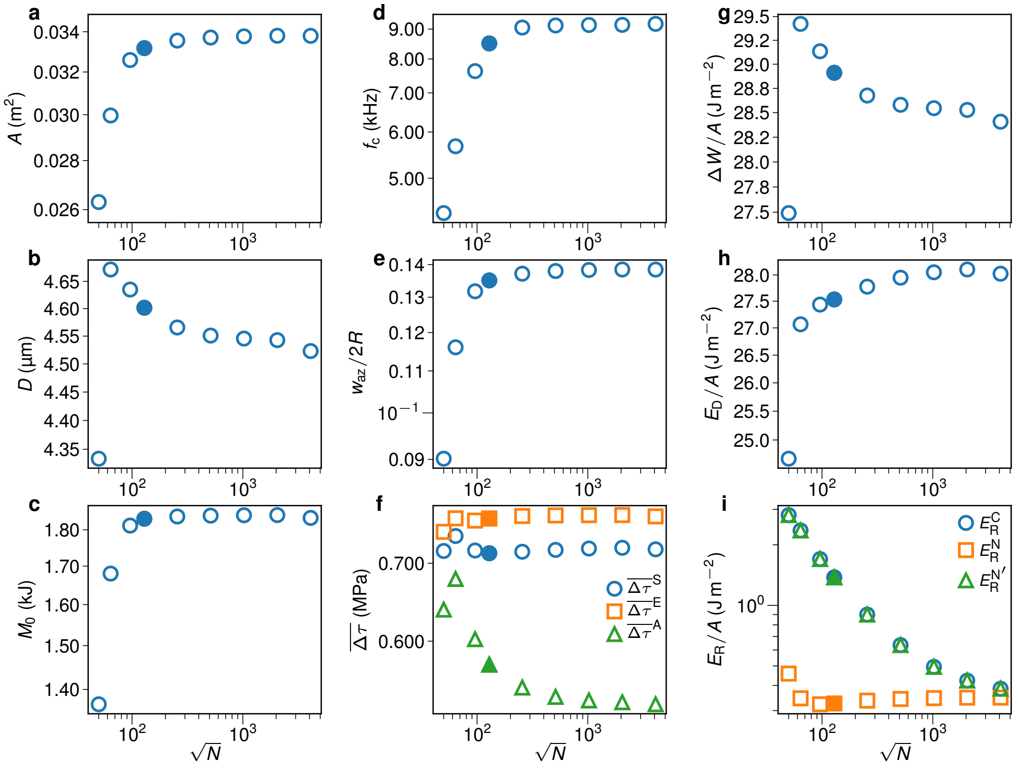

Figure S1: Convergence of extracted parameters of the earthquake rupture in $\chi = 2^{-2}$ model (identical in both scaling cases) with $\sqrt{N}$ =50, 64, 96, **128**, 256, 512, 1024, 2048, 4096. The filled marker indicates the selected element size ($\sqrt{N} = 128$) in this study. See Supplementary Note S1.

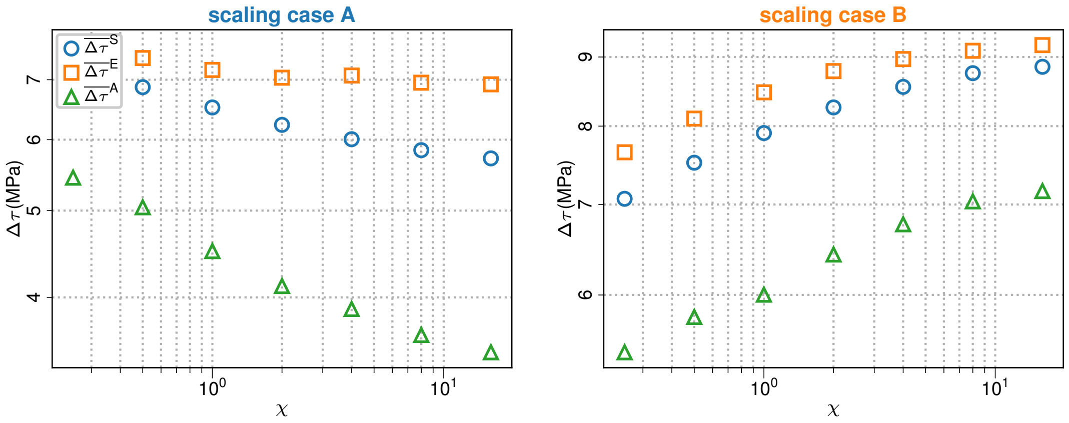

Figure S2: Averaged stress drop $\overline{\Delta\tau}$ estimated through different methods: $\overline{\Delta\tau}^{\mathrm{S}}$ (Eqn. S2), $\overline{\Delta\tau}^{\mathrm{E}}$ (Eqn. M7), and $\overline{\Delta\tau}^{\mathrm{A}}$ (Eqn. S3). See Supplementary Note S2.

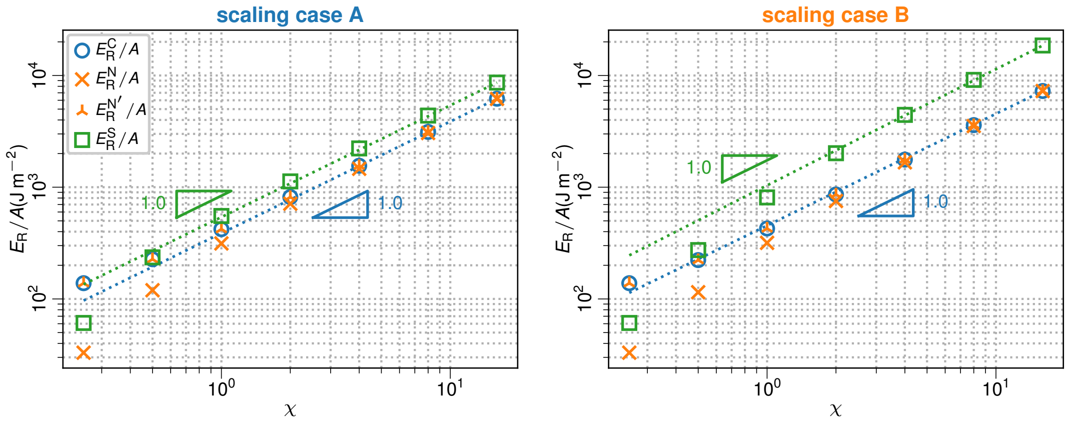

Figure S3: Radiated energy E_{R}/A estimated through different methods: $E_{\mathrm{R}}^{\mathrm{C}}$ (Eqn. M8), $E_{\mathrm{R}}^{\mathrm{N}}$ (Eqn. M7; Eqn. S4), $E_{\mathrm{R}}^{\mathrm{N}'}$ (Eqn. S5), and $E_{\mathrm{R}}^{\mathrm{S}}$ (Eqn. S6). See Supplementary Note S3.

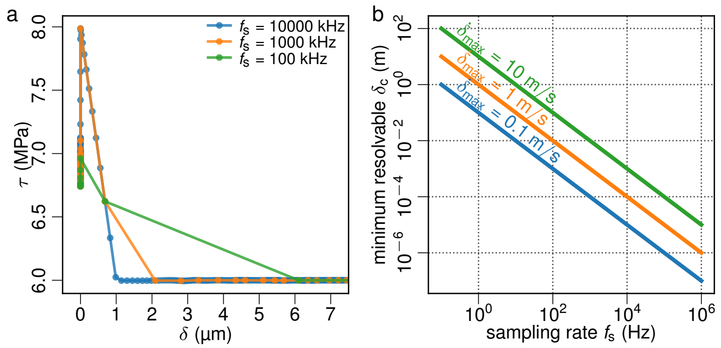

Figure S4: Effect of sampling rate f_s on apparent fault constitutive law. (a) The evolution of $\tau(\delta)$ from the $\chi = 1$ model in scaling case A at $(x, y, z) = (0.2, 0, 0.2)$ m measured at different f_s. Each dot represents an individual measurement. The measurement location is within the plateau region but outside the seed crack. Evidently, low f_s could make δ_c appear to be larger. (b) The minimum resolvable δ_c for given f_s and $\dot{\delta}_{max}$ given by Eqn. S8. See Supplementary Note S4.

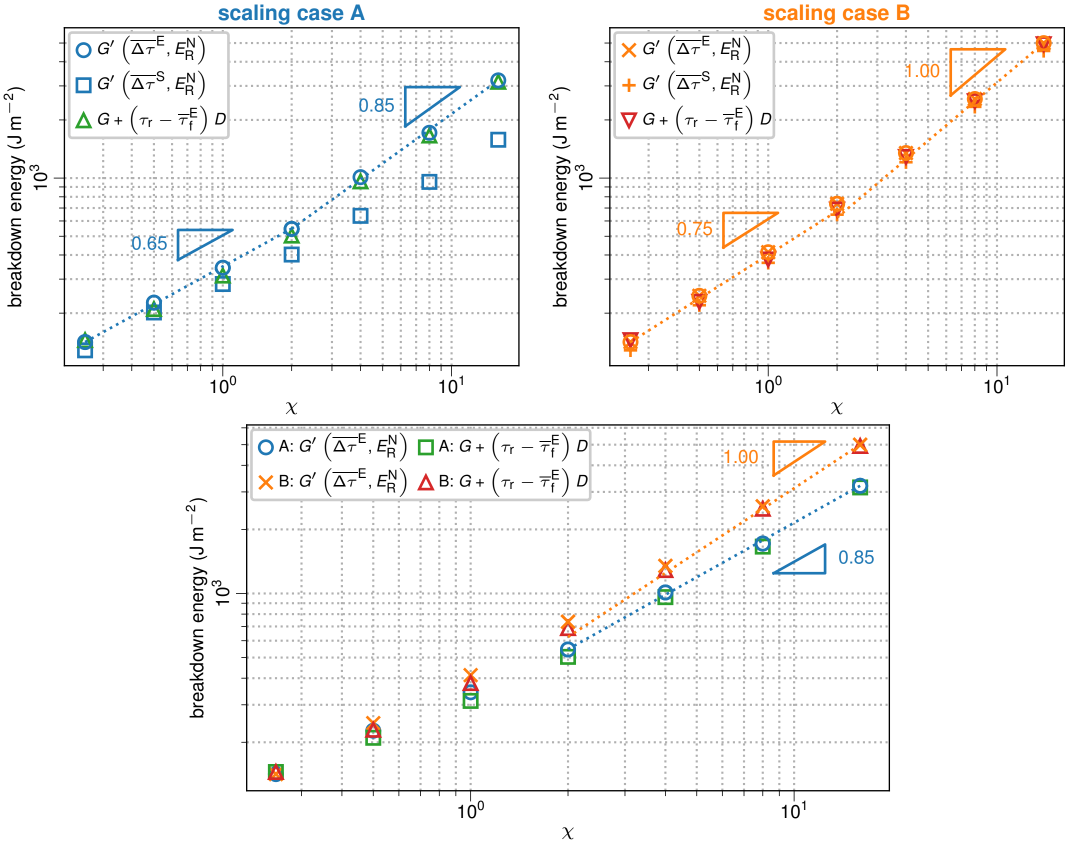

Figure S5: Breakdown energy G' estimated by different methods (see Methods).

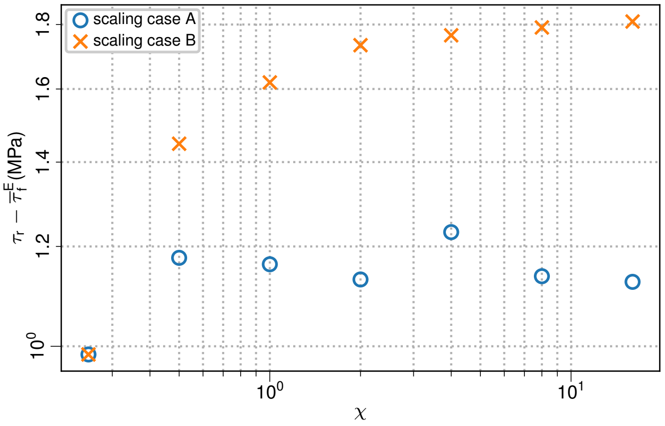

Figure S6: Averaged stress overshoot of each model $\left(\tau_{\mathrm{r}} - \overline{\tau}_{\mathrm{f}}^{\mathrm{E}}\right)$ is nearly scale-invariant. Overshoot is somewhat larger for the more abrupt arrest of scaling case B and somewhat smaller for the more gradual arrest of scaling case A.

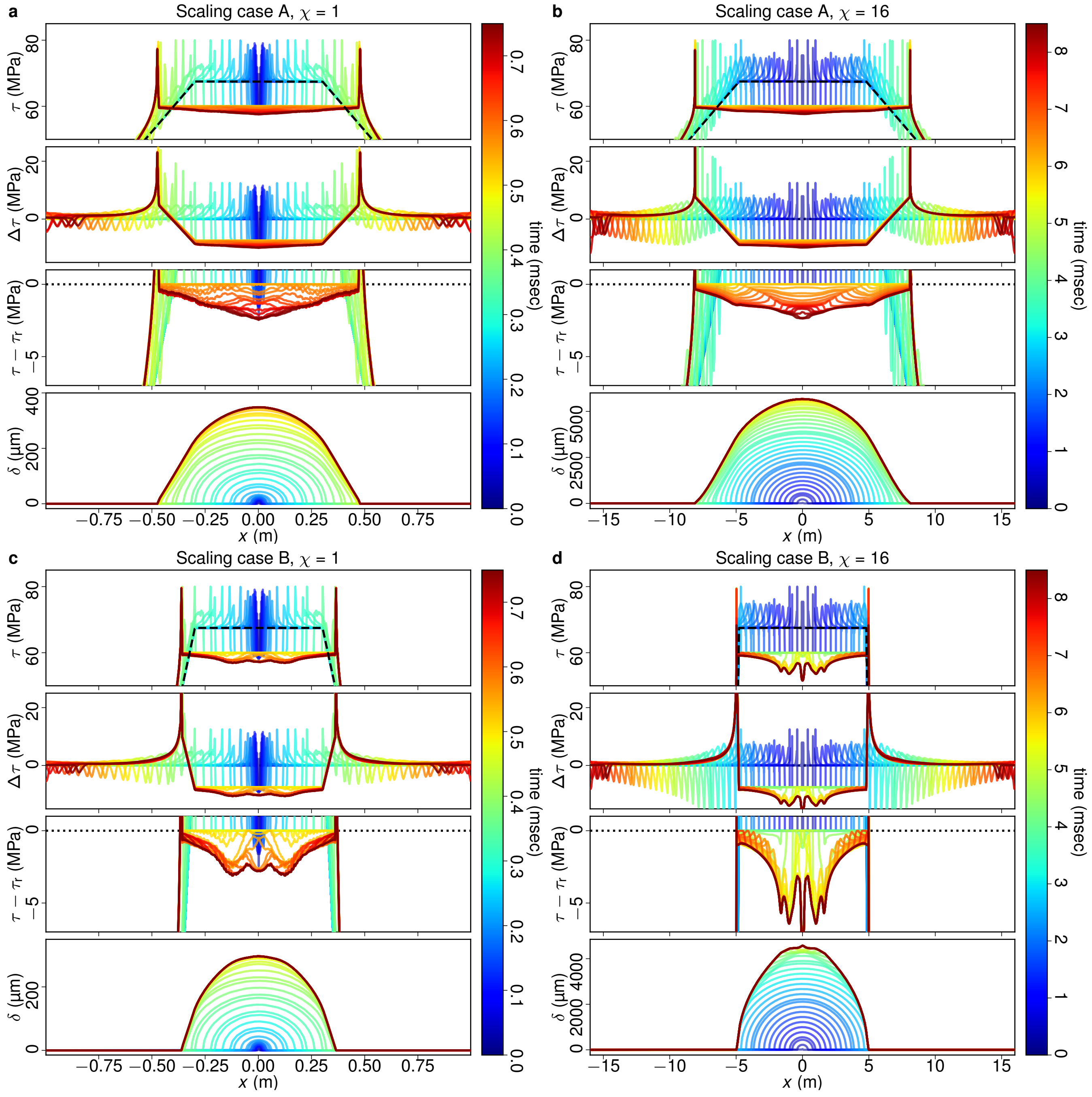

Figure S7: Snapshots of numerical simulations at $y = z = 0$. (a-d) Models of $\chi = 2^0$ and $\chi = 2^3$ in scaling case A and B, respectively. The $\tau - \tau_\mathrm{r}$ panel shows that the amplitude of stress overshoot correlates with the amplitude of slip δ, mainly due to the distance from the arrest location. There seem to be two arrest fronts, one comes from the arrest of the rupture front in the mode-II direction and the other one comes from the mode-III arrest.

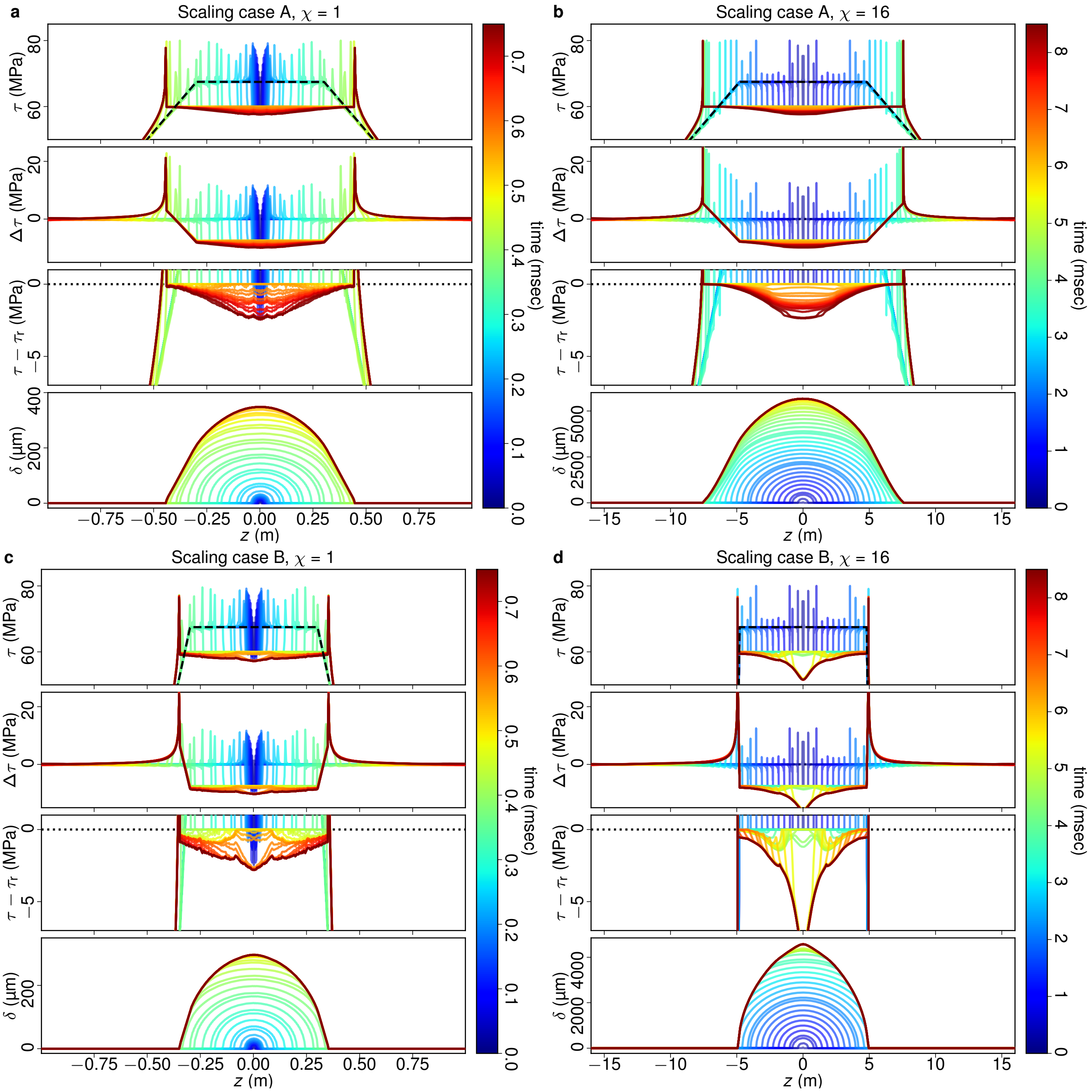

Figure S8: Snapshots of the same numerical simulations shown in Fig. S7, but at $x = y = 0$.

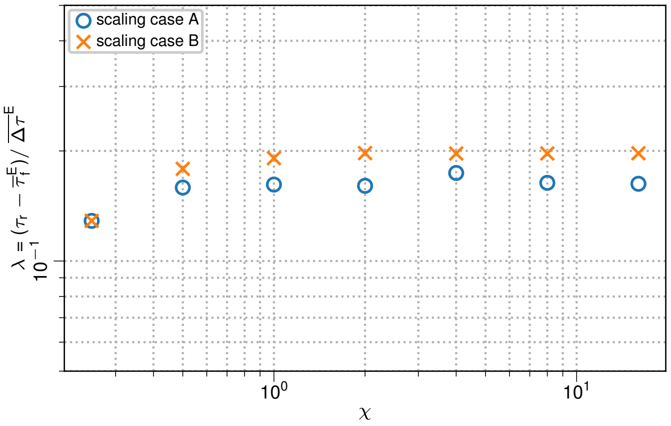

Figure S9: Parameter λ defined in ref.[1]: $\lambda \equiv (\tau_r - \tau_f)/\overline{\Delta\tau} = \overline{\Delta\tau}_{OS}/\overline{\Delta\tau}$, which is the averaged stress overshoot normalized by the averaged stress changes. Here we show $\lambda = \left(\tau_r - \overline{\tau}_f^E\right)/\overline{\Delta\tau}^E$ is nearly scale-invariant in both scaling cases and spans from 13% to 17% and from 13% to 20% for scaling case A and B, respectively.

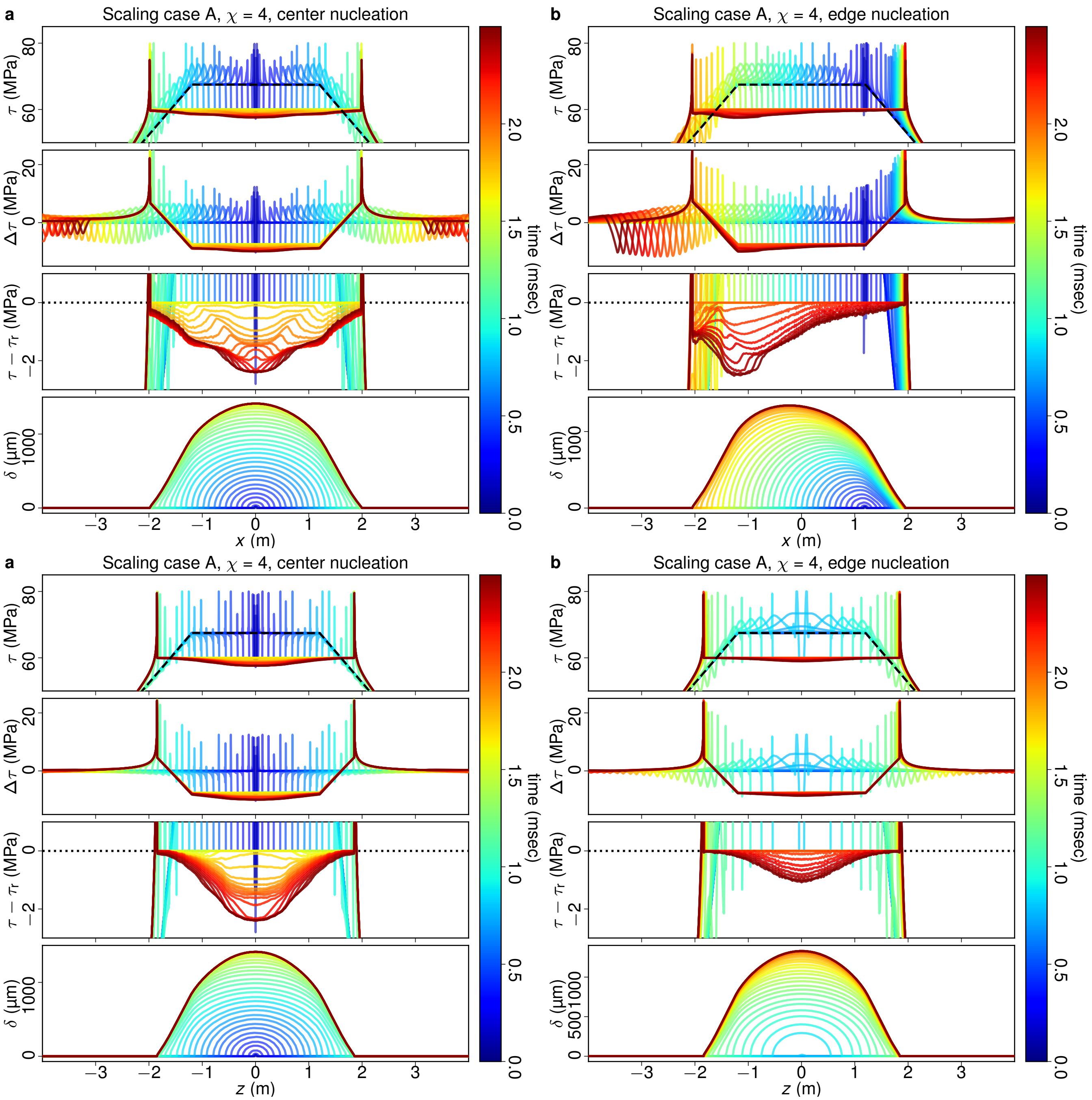

Figure S10: Comparison between center nucleation (a, c) and edge nucleation (b, d) models at $\chi = 4$. (a, b) Cross sections of the 3D model at $z = 0$, (c, d) cross sections of the same models at $x = 0$. For the edge nucleation, the rupture starts at $(x, y, z) = (0.7a, 0, 0.7a)$, where $r = 0.99a$.

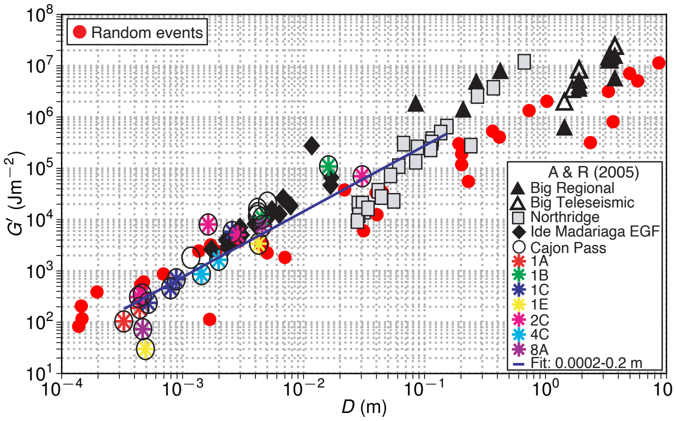

Figure S11: Randomly generated pseudo-earthquakes overlaid on data used in ref.[1], Fig. 8. Red circles are 50 randomly generated earthquake events that obey self-similarity and are uniformly distributed over $-2 \leq M_{\rm w} \leq 8$ with magnitude-independent overshoot -1 MPa $\leq \overline{\Delta\tau}_{\rm OS} \leq 2$ MPa. 30% of the events are not shown since $G' \leq 0$. G' of random events are computed by $G' = G + \overline{\Delta\tau}_{\rm OS} D$, where G is assumed to be negligible ($G = 1$ Jm^{-2}). While the observed scaling is not an exact match, we argue that it is within the uncertainty of the data.

References

1. Abercrombie, R. E. & Rice, J. R. Can observations of earthquake scaling constrain slip weakening? *Geophysical Journal International* **162**, 406–424 (2005).

2. Ke, C.-Y., McLaskey, G. C. & Kammer, D. S. Rupture Termination in Laboratory-Generated Earthquakes. *Geophysical Research Letters* **45**, 12784–12792 (2018).

3. Ke, C.-Y., McLaskey, G. C. & Kammer, D. S. The earthquake arrest zone. *Geophysical Journal International* **224**, 581–589 (2021).

4. Andrews, D. J. Rupture velocity of plane strain shear cracks. *Journal of Geophysical Research* **81**, 5679 (1976).

5. Palmer, A. C. & Rice, J. R. The Growth of Slip Surfaces in the Progressive Failure of Over-Consolidated Clay. *Proceedings of the Royal Society A: Mathematical, Physical and Engineering Sciences* **332**, 527–548 (1973).

6. Eshelby, J. D. The determination of the elastic field of an ellipsoidal inclusion, and related problems. *Proceedings of the Royal Society of London. Series A. Mathematical and Physical Sciences* **241**, 376–396 (1957).

7. Noda, H. & Lapusta, N. On averaging interface response during dynamic rupture and energy partitioning diagrams for earthquakes. *Journal of Applied Mechanics, Transactions ASME* **79**, 1–12 (2012).

8. Kostrov, V. & Riznichenko, V. Seismic moment and energy of earthquakes, and seismic flow of rock. *International Journal of Rock Mechanics and Mining Sciences & Geomechanics Abstracts* **13**, A4 (1976).

9. Ripperger, J., Ampuero, J.-P., Mai, P. M. & Giardini, D. Earthquake source characteristics from dynamic rupture with constrained stochastic fault stress. *Journal of Geophysical Research: Solid Earth* **112**, 1–17 (2007).

10. Singh, S. K. & Ordaz, M. Seismic energy release in Mexican subduction zone earthquakes. *Bulletin - Seismological Society of America* **84**, 1533–1550 (1994).

11. Perry, S. M., Lambert, V. & Lapusta, N. Nearly Magnitude-Invariant Stress Drops in Simulated Crack-Like Earthquake Sequences on Rate-and-State Faults with Thermal Pressurization of Pore Fluids. *Journal of Geophysical Research: Solid Earth* **125** (2020).

12. Guatteri, M. & Spudich, P. What Can Strong-Motion Data Tell Us about Slip-Weakening Fault-Friction Laws? *Bulletin of the Seismological Society of America* **90**, 98–116 (2000).
